# Metastatic Cutaneous Apocrine Carcinoma: Diagnostic and Therapeutic Challenges in a Rare Malignancy

**DOI:** 10.7759/cureus.113113

**Published:** 2026-07-21

**Authors:** Bryan L Chan, Chika Iguh, Andrew Hwang, Charity Huang

**Affiliations:** 1 Hematology-Oncology, Harbor-UCLA Medical Center, Torrance, USA; 2 Pathology, Harbor-UCLA Medical Center, Torrance, USA

**Keywords:** adnexal malignancy, androgen receptor (ar) expression, cutaneous apocrine carcinoma, hormone receptor positivity, immune checkpoint inhibition

## Abstract

Cutaneous apocrine carcinoma is a rare adnexal malignancy with limited evidence guiding treatment in the metastatic setting. We present the case of a 50-year-old man with hormone receptor-positive metastatic cutaneous apocrine carcinoma of the right axilla initially treated with surgical excision, nodal resection, and adjuvant radiation therapy. He later developed metastatic recurrence involving the spine and lungs, requiring surgical stabilization. Molecular profiling demonstrated a KMT2C mutation, microsatellite-stable disease, low tumor mutational burden, and overexpression of ERBB2 (Erb-B2 receptor tyrosine kinase 2/HER2), ERBB3 (Erb-B3 receptor tyrosine kinase 3/HER3), NFKB1 (nuclear factor kappa B subunit 1), CCND1 (cyclin D1), RET (RET proto-oncogene receptor tyrosine kinase), and AR (androgen receptor) pathways, while immunohistochemistry showed strong estrogen receptor positivity, androgen receptor expression, and HER2 negativity (unusual given the ERBB2 positivity). The patient later received sequential therapy with tamoxifen, pembrolizumab, androgen deprivation therapy, and, most recently, palliative radiation, followed by carboplatin/paclitaxel for progressive disease complicated by spinal cord compression. His clinical course was further complicated by severe neutropenia, radiation esophagitis, and chemotherapy-induced peripheral neuropathy. This case highlights the therapeutic complexity of metastatic cutaneous apocrine carcinoma and demonstrates the potential for meaningful disease control with endocrine and immune-based therapies despite progression through multiple lines of treatment. Given the absence of a standard treatment paradigm, more investigation in larger cohorts is needed to validate the role of these treatment approaches in improving outcomes in metastatic apocrine carcinoma.

## Introduction

Cutaneous adnexal carcinomas, including apocrine carcinoma, are rare solid tumor malignancies arising from sweat gland structures and account for a small fraction of cutaneous neoplasms. Primary cutaneous apocrine carcinoma most commonly occurs in regions rich in apocrine glands, such as the axilla, but can arise in other anatomic locations and exhibits a wide spectrum of clinical behavior ranging from indolent local disease to aggressive metastatic progression [1-3]. Due to its rarity (accounting for approximately 0.005 per 100,000 patients per year), management is largely guided by case reports and small retrospective series, with surgical resection remaining the standard treatment for localized disease [2]. Unfortunately, in the metastatic setting, no established systemic therapy options exist. In fact, despite frequent androgen receptor (AR) and occasional hormone receptor expression, evidence supporting endocrine-based treatment remains limited to case reports and small retrospective series. Many tumors demonstrate expression of estrogen receptor (ER), progesterone receptor (PR), and AR, providing a rationale for endocrine- and AR-directed therapies; however, reported responses from the aforementioned case reports and small retrospective series are often inconsistent and lack clinical durability [4,5].

We present a case of metastatic hormone receptor-positive cutaneous apocrine carcinoma that progressed through multiple lines of systemic therapy, including hormone-directed treatment and immunotherapy, and was ultimately complicated by significant neurologic sequelae requiring palliative radiation therapy and salvage chemotherapy. This case highlights the therapeutic challenges associated with advanced disease and underscores the need for more effective systemic treatment strategies for this uncommon malignancy.

## Case presentation

**Figure 1:**
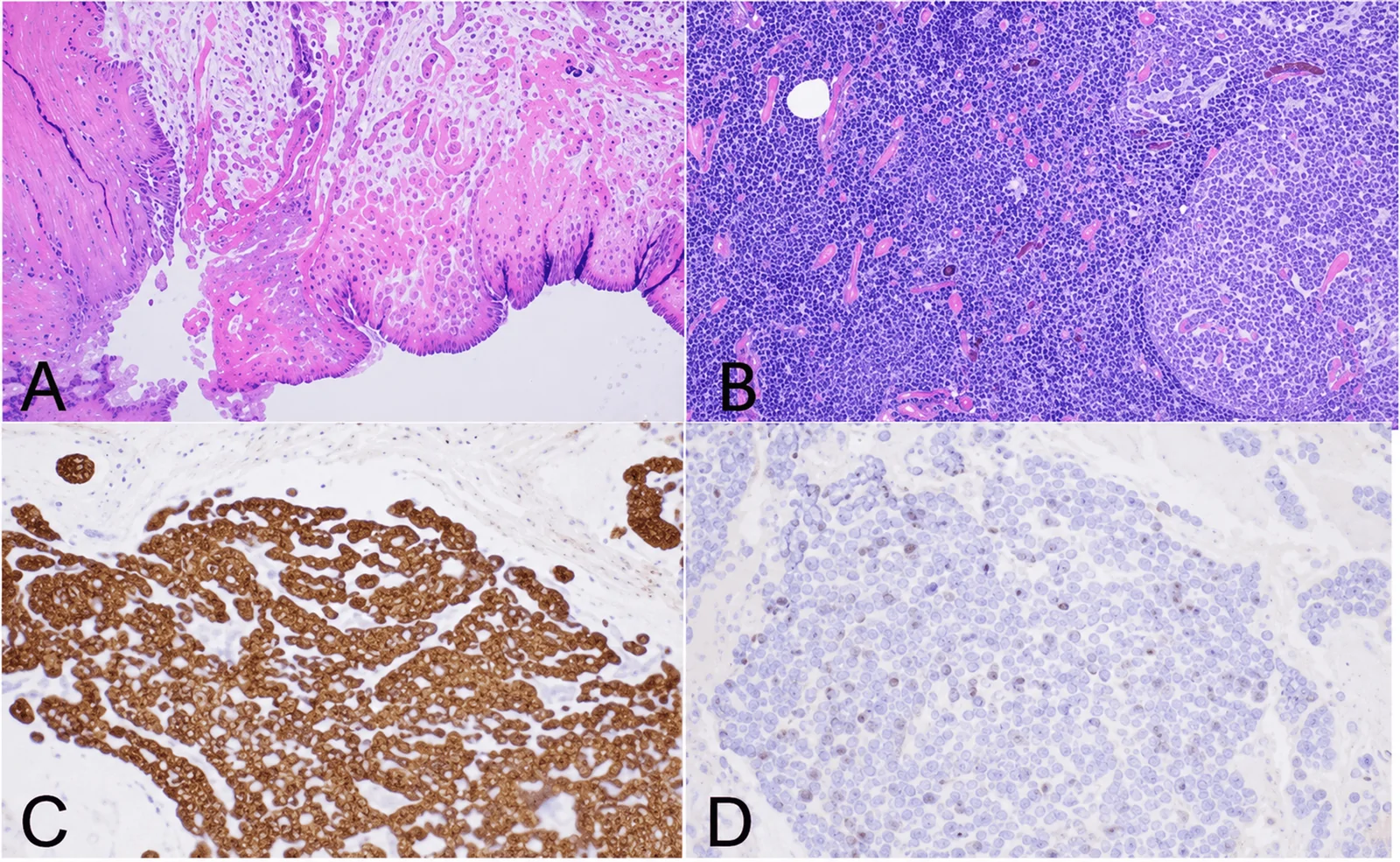
Right axilla skin resection (primary tumor) A: H/E stain, 20x, (+) AE1/AE3, GATA-3; B: H/E stain, 100x, (+) GATA-3, CAM 5.2; C: IHC stain, 100x, (+) cytokeratin AE1/AE3; D: IHC stain, 100x, (+) ER, mammaglobin

A 50-year-old man initially presented to his primary care physician in November 2020 with a painless soft tissue mass in the right axilla. He subsequently underwent surgical excision in December 2020, with histopathology demonstrating cutaneous apocrine carcinoma, a rare adnexal malignancy (Figure 1).

**Figure 2:**
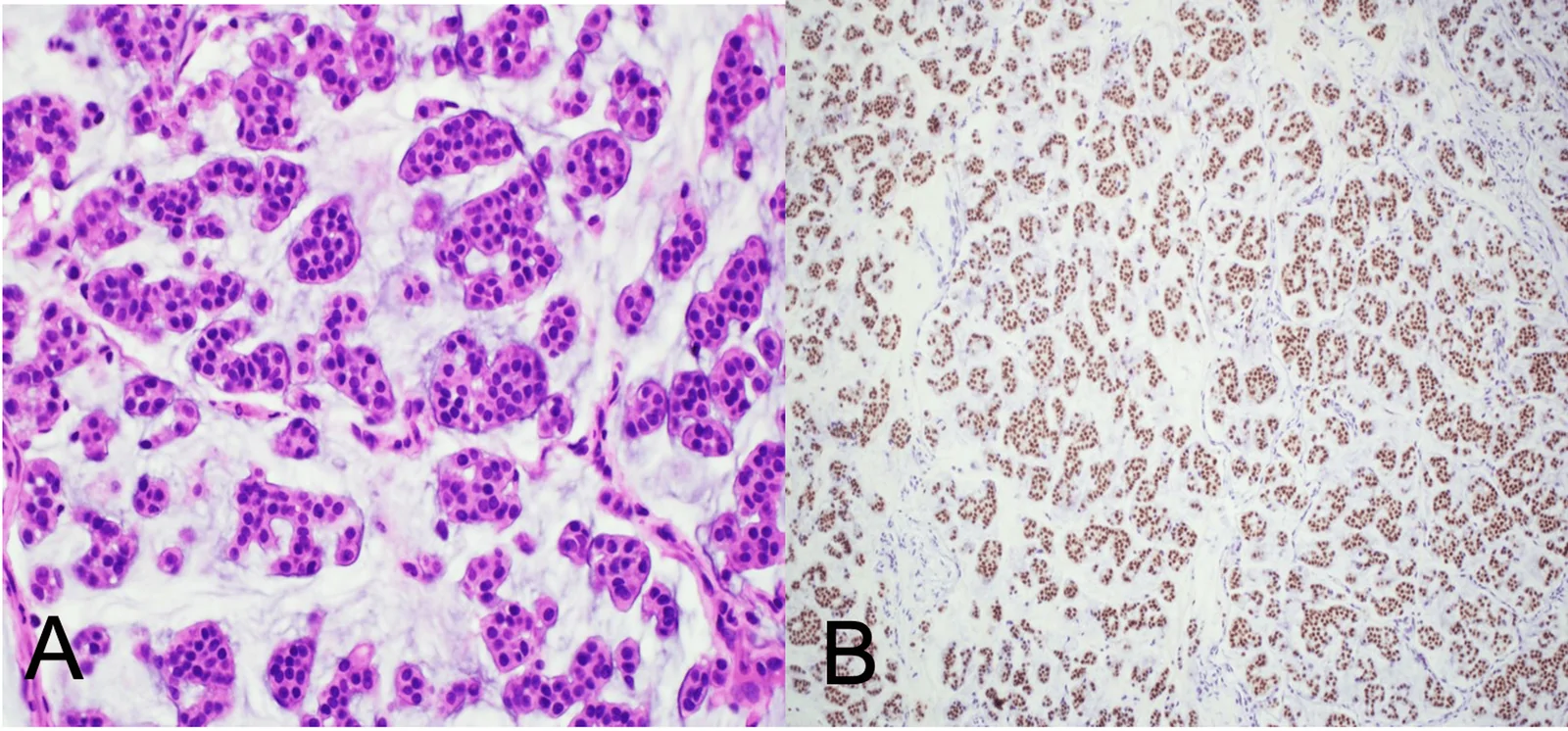
L1 corpectomy biopsy sample A: H/E stain, 400x, (+) GATA-3, CK-19; B: IHC stain, 100x, (+) PR, GATA-3, ER

He underwent re-resection of an involved lymph node in February 2021, followed by adjuvant radiation therapy. He remained in clinical remission until May 2023, when he was hospitalized for progressively worsening back pain. Imaging at that time demonstrated lesions concerning for metastatic disease involving both the spine and lungs. He underwent L1 corpectomy with T11-L3 posterior spinal fusion, and pathologic evaluation confirmed metastatic apocrine carcinoma (Figure 2). 

Comprehensive molecular profiling was performed. Next-generation sequencing identified a KMT2C (MLL3) mutation, a tumor mutational burden of 4.7 mutations per megabase, and microsatellite-stable disease. RNA sequencing demonstrated overexpression of ERBB2 (Erb-B2 receptor tyrosine kinase 2/HER2), ERBB3 (Erb-B3 receptor tyrosine kinase 3/HER3), NFKB1 (nuclear factor kappa B subunit 1), CCND1 (cyclin D1), RET (RET proto-oncogene receptor tyrosine kinase), and AR. Immunohistochemical analysis showed strong ER positivity (>90%), AR expression (3+), and HER2 negativity (1+).

Given the high level of ER expression, the patient was initiated on endocrine therapy with tamoxifen 20 mg daily between June 2023 and December 2023. However, disease progression was observed in December 2023 (progression-free survival (PFS) of six months). He was subsequently treated with pembrolizumab, a decision made individually, given reported prior success with immune checkpoint inhibitors in subsequent line settings in the literature, despite the patient's low TMB status and microsatellite-stable disease. His disease control lasted from December 2023 through November 2024 (PFS of 11 months). Zoledronic acid was added in February 2024 for the management of osseous metastatic disease. Repeat imaging in December 2024 demonstrated further progression, and the patient was subsequently started on androgen deprivation therapy with leuprolide and bicalutamide, given AR positivity. Despite the patient's "HER2-low" status, antibody-drug conjugates (ADCs) have not been regularly attempted in the literature, perhaps due to their novelty and cost, in addition to the lack of evidence. Given that the patient was enrolled at a county hospital system with limited access to these novel agents, ADCs were not attempted in this patient. 

**Figure 3:**
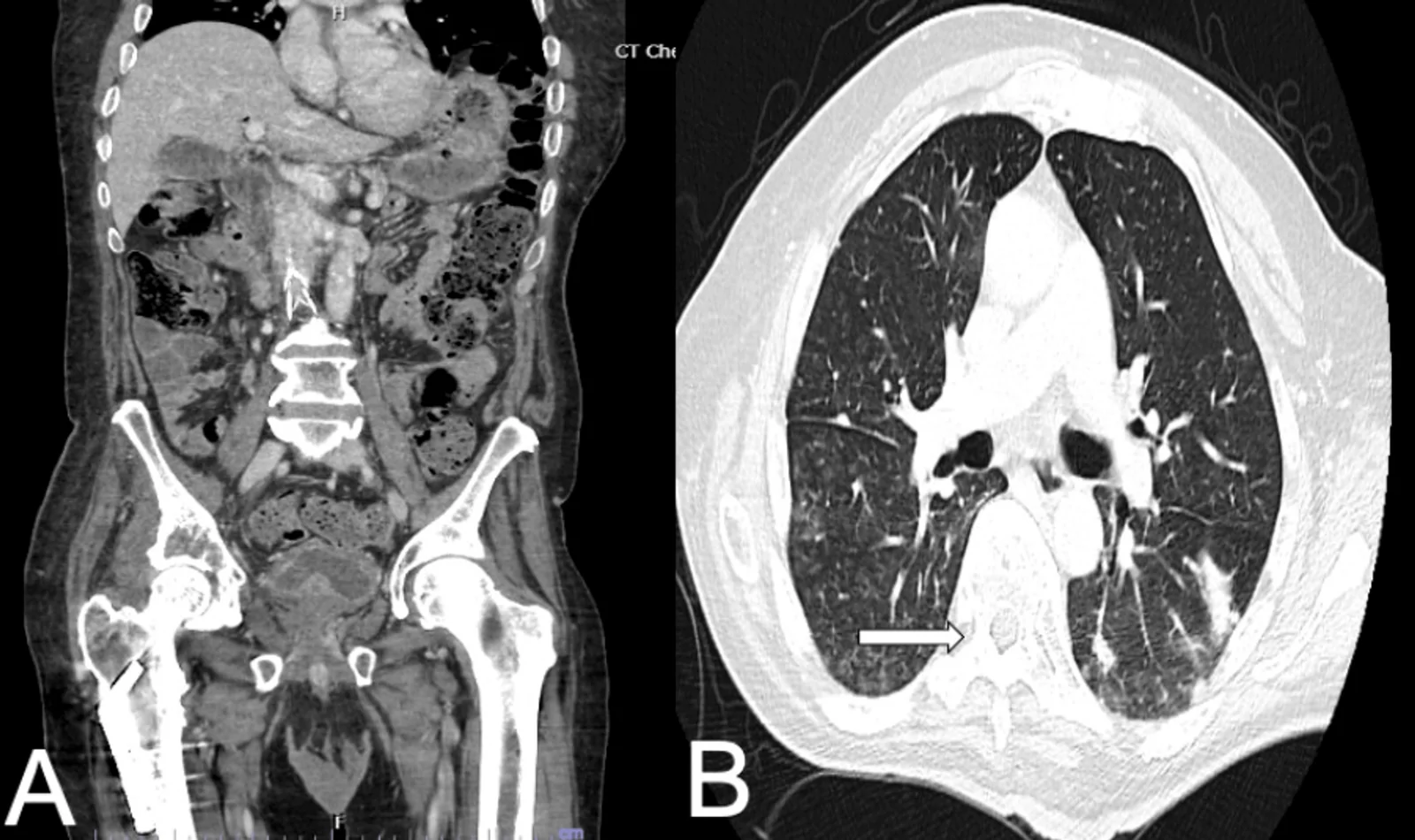
CT chest/abdomen/pelvis with intravenous (IV) contrast from the August 2025 admission A: Coronal slice of CT of the chest, abdomen, and pelvis; B: Axial slice of CT of the chest, with the white arrow indicating thoracic spinal cord compression.

In August 2025, the patient was admitted with altered mental status and progressive bilateral proximal lower extremity weakness, more pronounced on the right. Imaging revealed disease progression with severe thoracic spinal cord compression (Figure 3). He underwent palliative radiation therapy to T1-T5 (20 Gy in five fractions) and then received sequential therapy with inpatient chemotherapy with carboplatin (AUC 6) and paclitaxel (175 mg/m²). His hospital course was complicated by radiation-induced esophagitis and severe neutropenia requiring granulocyte colony-stimulating factor support and prolonged hospitalization.

Following discharge, he completed three additional cycles of carboplatin and paclitaxel (four cycles total) before discontinuing therapy due to the development of severe peripheral neuropathy in November 2025. The patient was previously undergoing evaluation for potential enrollment in clinical trials as of December 2025, but was unable to find a suitable trial given poor performance status and worsening debility despite months of outpatient physical rehabilitation. The patient passed away peacefully at home in June 2026.

## Discussion

Cutaneous apocrine carcinoma is an exceedingly rare malignancy arising from apocrine sweat glands, most commonly involving the axilla with a noted male predominance [2]. Diagnosis can be particularly challenging in poorly differentiated cases, as histologic and immunophenotypic overlap with other types of carcinoma (particularly breast) is common. Accurate diagnosis therefore requires careful clinicopathologic correlation, including integration of immunohistochemistry and clinical history. In our patient, strong ER (>90%) and AR positivity supported the diagnosis and are consistent with immunoprofiles previously reported in medical literature, highlighting the potential relevance of hormone-driven biology in this disease [3].

The management of advanced cutaneous apocrine carcinoma remains poorly defined due to its rarity and the absence of prospective clinical trials. For localized disease, wide surgical excision with or without adjuvant radiation remains the standard of care. However, in the metastatic setting, treatment strategies are largely extrapolated from breast cancer and other adenocarcinomas, often with limited success [3,4]. Hormonal therapies targeting estrogen and androgen pathways have demonstrated variable and frequently transient responses, suggesting underlying tumor heterogeneity and the potential for early resistance mechanisms [5]. Similarly, immune checkpoint inhibitors have shown anecdotal activity in select patients, particularly in tumors with low tumor mutational burden and microsatellite stability. Cytotoxic chemotherapy, including platinum and taxane-based regimens, continues to be commonly utilized for progressive disease, but responses are often short-lived and accompanied by significant toxicity, which may limit treatment durability [6]. Emerging molecular data, including alterations such as KMT2C mutations and overexpression of pathways involving ERBB family members and AR signaling, suggest potential avenues for targeted therapy; however, these approaches remain investigational and are not yet supported by robust clinical evidence [7,8].

Historically, advanced disease has been associated with poor outcomes, largely derived from small case series and retrospective reports, which suggest limited responsiveness to conventional cytotoxic chemotherapy and an overall unfavorable prognosis [1,6]. Due to its rarity, precise estimates of median overall survival (OS) in metastatic cutaneous apocrine carcinoma are not well established (with recent cohorts stating a median OS of 29 months); however, available reports consistently describe aggressive clinical behavior once distant metastases develop, with relatively short durations of disease control on systemic therapy [9].

In this context, the clinical course of our patient is notable. Despite initial diagnosis in 2020 and metastatic recurrence in 2023, he remains alive following multiple lines of systemic therapy, including tamoxifen (five months), pembrolizumab (11 months), and androgen deprivation therapy with AR pathway inhibition for approximately eight to nine months. These treatment durations suggest meaningful clinical benefit rather than treatment-refractory disease. Notably, despite the aforementioned median OS of 29 months for the typical patient with this malignancy, this patient survived for over five-and-a-half years from initial diagnosis (November 2020 to June 2026) and three years from the development of distant metastases (May 2023 to June 2026), truly emphasizing the efficacy of the sequential treatment approach. Importantly, apocrine carcinomas frequently express AR, providing a biologic rationale for endocrine-based approaches, while emerging reports have also described responses to immune checkpoint inhibition in selected patients [8,9]. Table 1 presents the reported systemic therapies for unresectable or metastatic cutaneous apocrine carcinoma.

**Table 1:** Reported systemic therapies for unresectable or metastatic cutaneous apocrine carcinoma ORR: overall response rate; PFS: progression-free survival

Therapy class	Representative regimen	Patient selection	Best reported outcome	Key Reference
Chemotherapy	Platinum, taxane, S-1	Unselected	ORR 22-37% (multicenter cohort)	Saito et al. [7], 2025
Endocrine therapy	Tamoxifen	ER-positive	Isolated reports; no established ORR/PFS	Tsuruta et al. [10], 2024 (review)
Anti-androgen	Bicalutamide ± LHRH agonist	AR-positive	Durable response in the case report	Collette et al. [9], 2020
HER2-directed	Trastuzumab + pertuzumab + docetaxel	HER2-positive	Major response permitting surgery	Otsuka et al. [11], 2016
Immunotherapy	Pembrolizumab or nivolumab	PD-L1-positive/unknown	Durable response in isolated cases	Rogatsch et al. [12], 2018; Murayama et al. [13], 2025

Overall, this case illustrates the complexities and therapeutic limitations of metastatic cutaneous apocrine carcinoma. Given the lack of standardized treatment and poor durability of existing options, early referral for clinical trial enrollment and exploration of biomarker-driven targeted strategies are recommended [9]. Collaborative efforts and accumulation of real-world data are needed to better define optimal management and improve outcomes in this rare malignancy.

## Conclusions

Cutaneous apocrine carcinoma is an exceedingly rare malignancy with strong male predominance and diagnostic challenges requiring clinicopathologic correlation. This case highlights the complex nature of metastatic cutaneous apocrine carcinoma. Rather than demonstrating uniformly aggressive or refractory disease, this case highlights the potential for prolonged disease control with sequential targeted and immunotherapeutic strategies in metastatic cutaneous apocrine carcinoma. The patient's survival beyond what is typically expected based on historical reports underscores the heterogeneity of this disease and suggests that selected patients may achieve durable benefit from AR-directed and immune-based therapies. Particularly, given the absence of a standard treatment paradigm, further investigation in larger cohorts is needed to better define prognostic factors and to validate the role of these treatment approaches in improving outcomes in metastatic apocrine carcinoma. In addition, the routine use of multidisciplinary molecular tumor boards when managing rare, non-standardized malignancies would be a valuable asset to future cohorts tasked with treating these challenging and unique cases.
